# *In silico* identification of essential proteins in *Corynebacterium pseudotuberculosis* based on protein-protein interaction networks

**DOI:** 10.1186/s12918-016-0346-4

**Published:** 2016-11-04

**Authors:** Edson Luiz Folador, Paulo Vinícius Sanches Daltro de Carvalho, Wanderson Marques Silva, Rafaela Salgado Ferreira, Artur Silva, Michael Gromiha, Preetam Ghosh, Debmalya Barh, Vasco Azevedo, Richard Röttger

**Affiliations:** 1 Department of General Biology, Instituto de Ciências Biológicas (ICB), Federal University of Minas Gerais (UFMG), Belo Horizonte, Brazil; 2 Institute of Biological Sciences, Federal University of Para, Belém, PA Brazil; 3 Biotechnology Center (CBiotec), Federal University of Paraiba (UFPB), João Pessoa, Brazil; 4 Department of Biochemistry and Immunology, Federal University of Minas Gerais (UFMG), Belo Horizonte, Brazil; 5 Department of Biotechnology, Indian Institute of Technology (IIT) Madras, Tamilnadu, India; 6 Department of Computer Science, Virginia Commonwealth University, Richmond, VA USA; 7 Centre for Genomics and Applied Gene Technology, Institute of Integrative Omics and Applied Biotechnology (IIOAB), Nonakuri, Purba Medinipur, West Bengal India; 8 Department of Mathematics and Computer Science, University of Southern Denmark, Odense, Denmark

**Keywords:** Protein-protein interaction network, Essential proteins, *Corynebacterium pseudotuberculosis*

## Abstract

**Background:**

*Corynebacterium pseudotuberculosis* (*Cp*) is a gram-positive bacterium that is classified into *equi* and *ovis* serovars. The serovar *ovis* is the etiological agent of caseous lymphadenitis, a chronic infection affecting sheep and goats, causing economic losses due to carcass condemnation and decreased production of meat, wool, and milk. Current diagnosis or treatment protocols are not fully effective and, thus, require further research of *Cp* pathogenesis.

**Results:**

Here, we mapped known protein-protein interactions (PPI) from various species to nine *Cp* strains to reconstruct parts of the potential *Cp* interactome and to identify potentially essential proteins serving as putative drug targets. On average, we predict 16,669 interactions for each of the nine strains (with 15,495 interactions shared among all strains). An *in silico* sanity check suggests that the potential networks were not formed by spurious interactions but have a strong biological bias. With the inferred *Cp* networks we identify 181 essential proteins, among which 41 are non-host homologous.

**Conclusions:**

The list of candidate interactions of the *Cp* strains lay the basis for developing novel hypotheses and designing according wet-lab studies. The non-host homologous essential proteins are attractive targets for therapeutic and diagnostic proposes. They allow for searching of small molecule inhibitors of binding interactions enabling modern drug discovery. Overall, the predicted *Cp* PPI networks form a valuable and versatile tool for researchers interested in *Corynebacterium pseudotuberculosis*.

**Electronic supplementary material:**

The online version of this article (doi:10.1186/s12918-016-0346-4) contains supplementary material, which is available to authorized users.

## Background

*Corynebacterium pseudotuberculosis* (*Cp*) belongs to the supra generic CMNR group (*Corynebacterium, Mycobacterium, Nocardia, Rhodococcus*) of bacteria [1]. It is an intracellular Gram-positive pathogenic bacterium that is fimbriated, non-motile and non-capsulated [2] and is present in two serovars: *ovis* and *equi* [3]. The serovar *equi* infects mainly horses and cattle while the serovar *ovis* is the etiological agent of caseous lymphadenitis (CLA), a chronic infectious disease affecting mainly sheep and goat populations. It can also infect humans upon occupational exposure [4, 5]. CLA is prevalent in several countries around the world [6–21] and causes significant economic losses due to low carcass quality, a decrease in the production of meat, wool and milk [22, 23], while also causing animal mortality due to suppurative meningoencephalitis [24]. The available methods for CLA diagnosis or treatment are not effective enough and require further research to tackle the threats posed by *C. pseudotuberculosis*. Hence, it becomes important to know how the genes, transcripts, proteins and other molecules inside the bacterial cells interact with each other and with the outer environment to perform their biological functions [25–29]. From this perspective, the study of proteins and their interactions allows for a better understanding of the molecular mechanism of cells at a system level [30, 31]. The protein-protein interactions (PPI) form a complex network represented as a graph, where the nodes represent proteins and undirected edges connecting these nodes represent the interactions between the proteins [32, 33]. Generally, PPI networks have shown to be a great vehicle for developing new hypotheses and designing novel laboratory experiments [34, 35]. Furthermore, essential proteins can be identified by topological analysis. An essential protein is defined as a gene which demonstrates to be lethal for the organism when subject to a knock-out [36]. Therefore, essential proteins are potential drug targets [37–41], enabling the development of new drugs against pathogenic microorganisms [42–45].

Generally, *in silico* reconstruction of biological networks is a long standing problem and is applied to various different types of networks. As prominent example may serve the reconstruction of the regulatory network of various different *Corynebacteria* which has become a widely used resource [46, 47].

In this manuscript, we predicted the potential PPI networks of nine strains of *Cp* serovar *ovis* using the interolog mapping method. The interolog mapping method was already successfully applied in several other studies, for example to predict the interactions in *Mycobacterium tuberculosis* [48], *Leishmania spp.* [49], mouse [50] and *Bacillus licheniformis* [51].

While Yu et al. [57] used an identity > 80 % in their “generalized interolog mapping” to transfer interactions, we have refined this cut-off in one of our previous studies by means of an exhaustive *in silico* evaluation [52]. We used the experimentally validated and manual curated small-scale interactions from the DIP database (Database of Interacting Proteins) [53] as the gold standard and further collected the interactions from three different and independent PPI databases (STRING (search tool for recurring instances of neighbouring genes) [54], IntAct [55] and PSIbase (database of Protein Structural Interactome map) [56]) as the input for the network transfer and aimed to reconstruct the interactions in the DIP database. In this setting we archived a specificity of 0.95, sensitivity of 0.83 and a precision of 0.99 when we compared our predictions with the gold standard [52].

In a different study, Yu et al. archived an accuracy of 54 % when employing a similar method for transferring the interactome from *C. elegans* to *S. cerevisiae* [57]; two evolutionarily rather different organisms. In this study, we are convinced that our predictions are more reliable as with *C. glutamicum* we have an exhaustively studied model organism at hand which is evolutionary very close to *Cp* [46, 47, 58].

Due to this exhaustive previous work, we only perform a brief *in silico* sanity check of the derived networks before identifying essential proteins which might be promising targets for further wet-lab experiments. It is important to note that the reported PPI networks are a mere list of potential interactions and should serve as a basis for further research. The experimental validation for the predicted potential interactome is out of the scope of this study.

## Results and discussion

### Prediction of *C. pseudotuberculosis* PPI network

For all nine strains of Cp, we predicted a total of 150,019 potential protein-protein interactions involving 10,370 of the in total 18,890 proteins (Table 1).

**Table 1:** Number of proteins and interactions for each serovar ovis strain

Strain	Proteome	Interacting proteins	Interactions	Reference
Cp1002	2,090	1,156	16,710	[10]
Cp267	2,148	1,164	16,728	[11]
Cp3995	2,142	1,141	16,600	[12]
Cp4202	2,051	1,148	16,712	[12]
CpC231	2,091	1,151	16,647	[10]
cpfrc	2,110	1,165	16,897	[13]
CpI19	2,095	1,158	16,715	[14]
CpP54B96	2,084	1,149	16,537	[9]
CpPAT10	2,079	1,138	16,473	[15]

Proteome: total number of proteins; Interacting proteins: number of proteins participating in the interaction network. Interactions: number of predicted interactions used for network composition.

The analysis of the prediction origin shows that the vast majority of interactions were mapped from phylogenetically close organisms, belonging to the genus *Corynebacterium* (in ~99 % of the cases) but also reveals some predictions from more distant organisms (Additional file 1: Figure S1).

### Validation of the network properties

As described above, in order to check the credibility of our network predictions, we performed statistical sanity checks on the network topology. We were able to show that the node degree distribution approximately follows a power-law distribution and in combination with shortest-path analysis, suggest that the predicted networks have a scale-free topology, both prevalent and relevant characteristics pertaining to biological networks. The clustering coefficient, correlation and regression analysis using the R-Squared values from predicted *Cp* interaction networks and the Shapiro-Wilk [59] normality test demonstrated that the degree distribution of predicted interaction networks do not follow a normal distribution (*p*-value < 2.2e-16) (Additional file 2). All analyses suggest that the networks were not formed by spurious interactions but originated due to a biological growth process. Moreover, the high Clustering Coefficient of the predicted networks suggest the existence of self-organization inside the biological cell motivated by the interactions [60]. Furthermore, we were able to confirm the existence of several clusters of our networks by means of literature research, increasing the confidence in the methodology and the predictions (Additional file 3). Please note, that these test comprise mere sanity checks of the potential networks and should not be misinterpreted as exhaustive proof for correctness of the potential interactions.

Not surprisingly, due to the extremely clonal life-style [61], almost all predicted interactions are found in all *Cp ovis* strains (i.e., core-interactome). Strain specific interactions or the accessory interactions are also of great interest as they might explain the biological specifics of a strain. However, here we focused on exploring the core-interactome of the nine *Cp ovis* strains aiming to better understand the serovar *ovis* in general and derive potential viable targets for further wet-lab research.

### Essential proteins

Essential proteins are proteins which have a lethal effect when removed from the organism. It was shown that the node degree of a protein (i.e., the number of interactions of that protein) is correlated with the lethality [62, 63]. Thus, potential essential genes may be identified by identifying hub nodes in the network, i.e., nodes with a very high node degree (refer to the Methods section for details).

In our predicted networks, we identified 181 hub proteins each having 68 or more interactions. In the set of hub proteins, we find proteins involved in biological processes related to carbon metabolism, cell envelope and cell wall, DNA metabolism, nucleotides biosynthesis, folding, translocation, ribosomal translation factors, tRNA synthetase, RNA metabolism and respiratory pathways, among others. Aiming to verify the essentiality of these *Cp* hubs, we searched for homologous proteins in the database of essential genes (DEG) [64, 65]. Among the 181 hub proteins, 180 had homologous counterparts already stored as essential in DEG, showing the effectiveness of our method for identifying the essential proteins (Additional file 4).

The DNA repair protein (RecN), was the only essential protein not found in DEG, apparently being exclusive to *Cp*. RecN is responsible for maintaining DNA integrity when exposed to various stress conditions. Despite the conserved mechanism, both metabolic pathways and proteins can differ in each species [66]. This indicates the essentiality of this protein and explains why there was no counterpart found in DEG.

Even though the vast majority of proteins have homologs in DEG, this does not reduce the importance of reporting their essentiality. Considering *Cp* is not covered by DEG till today, the description of essentiality in this organism is novel for all 181 proteins. It is worth noting that while most essential proteins have homologs from over 20 organisms covered by DEG, three proteins have homologs in only a single organism, demonstrating either the lack of experiments which would support their essentiality, the lack of protein conservation across different species or that the essentiality of these proteins is not conserved across species [67]. These proteins are: Catalase (KatA), Endonuclease III (Nth) and Trigger factor Tig (Tig). Catalase (KatA) is homologous to KatE from *Salmonella enterica*. KatA is an oxidoreductase enzyme which decomposes hydrogen peroxide (H_2_O_2_). It was already studied for instance in *C. glutamicum* [68, 69] and *C. pseudotuberculosis* [70]. Endonuclease III (Nth) has a homologous counterpart in *Haemophilus influenzae* stored in DEG. Nth is a base excision repair enzyme [71] that participates in a pathway preventing the loss of DNA functionality e.g., by spontaneous mutagenic lesion [72] or near-UV radiations [73]. This mechanism is well studied and is conserved in the *Corynebacterium* species [74]. Trigger factor Tig (Tig) has a DEG homology against *Pseudomonas aeruginosa* and participates in the protein folding process.

Additionally, in order to propose potential biomarkers or therapeutic targets among the essential proteins, a search for homologs in the host organisms *O. aries, C. hircus, B. taurus, E. caballus* and *H. sapiens* was performed. We identified 41 non-host homologous proteins, i.e., these are essential proteins of *Cp* which have no homologs in one or more host organisms. Among these non-host homologous proteins, 15 are with no alignment hits against any of the five hosts, nine with no alignment hits against *O. aries* and *C. hircus* and the remaining 17 had only low identity and low coverage hits (Additional file 5: Figure S2).

The 24 non-host homologous proteins without any significant hit against at least one host are: chorismate synthase (*aroC*), dihydrodipicolinate reductase (*dapB*), DNA primase (*dnaG*), elongation factor P (*efp*), cell division protein (*ftsZ*), ATP phosphoribosyl transferase (*hisG*), dihydroxy-acid dehydratase (*ilvD*), aspartate kinase (*lysC*), UDP-N-acetylglucosamine (*murA*), transcription anti-termination protein (*nusG*), uridylate kinase (*pyrH*), DNA repair protein (*recN*), transcription termination factor (*rho*), 50S ribosomal protein L1 (*rplA*), 50S ribosomal protein L10 (*rplJ*), 50S ribosomal protein L31 (*rpmE*), DNA-directed RNA polymerase subunit alpha (*rpoA*), 30S ribosomal protein S3 (*rpsC*), 30S ribosomal protein S6 (*rpsF*), 30S ribosomal protein S13 (*rpsM*), holliday junction DNA helicase subunit (*ruvA*), SsrA-binding protein/SmpB superfamily (*smpB*), indole-3-glycerol phosphate synthase (*trpC2*) and anthranilate synthase (*trpE*). As these proteins are essential to *Cp* but do not occur in the host organisms, they naturally are a potential drug-targets because inhibiting these proteins is likely to be lethal for *Cp* whereas the host proteome remains unaffected due to the missing homologs and furthermore due to the greater potential of these proteins to participate in inter-species interactions with the host [75].

A small subset of the essential non-host homologous proteins participates in the same metabolic pathway and thus is of particular interest. These proteins are the Indole-3-glycerol phosphate synthase (trpC2), Anthranilate phosphoribosyl transferase (trpD), Anthranilate synthase (trpE) and Anthranilate synthase component II (trpG); all are involved in the metabolic pathway of tryptophan biosynthesis, which produces amino acids of biotechnological interest and are essential in human and animal nutrition [76]. This metabolic pathway involves proteins encoded by the genes of the *Cp* operon trpABCDGEF which was already studied and characterized in other organisms [77]. Prephenate dehydratase (pheA) is involved in the metabolic pathway of phenylalanine biosynthesis from the chorismate pathway [78]. Tryptophan, phenylalanine and tyrosine are aromatic amino acids and share the beginning of a pathway found and characterized in *C. glutamicum* [79] whose proteins are also partially present in the *Cp* biovar *ovis*. Additionally, the other essential proteins interacting in this network are Tryptophanyl-tRNA synthetase (trpS), Phenylalanyl-tRNA synthetase subunit alpha (pheS) and Tyrosyl-tRNA synthetase (tyrS).

Furthermore, the cluster analysis draws attention to the *Cp* iron acquisition system, which is well characterized and contributes to the survival and virulence of microorganisms [80, 81]. The cluster consists of proteins associated with different iron acquisition systems, a strategy to acquire iron from multiple sources in low availability [82], suggesting both, alternative metabolic pathways and alternative proteins from different operons exerting the same function. In the potential *Cp* networks, these multiple systems interact with each other and consist mainly of proteins from the operons *fag*, *ciu, fec* and *hmu*, suggesting a potential ability to import iron from the host [83, 84] (Additional file 3)*.*

## Conclusions

For the first time, we reported potential PPI networks for nine *Cp ovis* strains based on an *in silico* prediction. The employed methodology is well-established and we consider this work as the starting-point for the development novel hypothesis and the design of upcoming wet-lab studies. Nevertheless, it is important to notice that the in silico predictions only represent a candidate list of potential interactions and may contain false-positives, in particular when considering that the original interactions utilized for the prediction also contain false-positives themselves.

The main contribution and analysis of this work is the identification of potentially essential genes which have a very high node-degree in the network reducing the impact of sporadic false-positives. In total, we identified 181 essential proteins, 41 of them being non-host homologous, hence becoming good candidates for drug development or CLA diagnosis. Since the essential proteins interact with many others, it is natural to assume they are associated with various biological processes, in their own species as well as in the host, and hence are attractive targets for therapeutic and diagnostic proposes [85]. Especially each predicted interaction of an essential protein is a potential candidate for the identification of inhibitors [86, 87] and thus opening several drug development opportunities targeting *C. pseudotuberculosis*. Especially the non-host homologous essential proteins might serve as potential targets for inhibiting interaction class drugs [40, 86, 88]. Generally, all reported potential interactions might allow for searching small molecule inhibitors of binding interactions [45, 86, 89], making modern drug discovery research possible [90]. By knowing the interaction partners of a protein, it is hence possible to provide a systemic view of the organism [91]. To sum up, the PPI networks reported here are valuable tools for researchers to identify proteins or interactions as potential drug targets.

## Methods

**Fig. 1:**
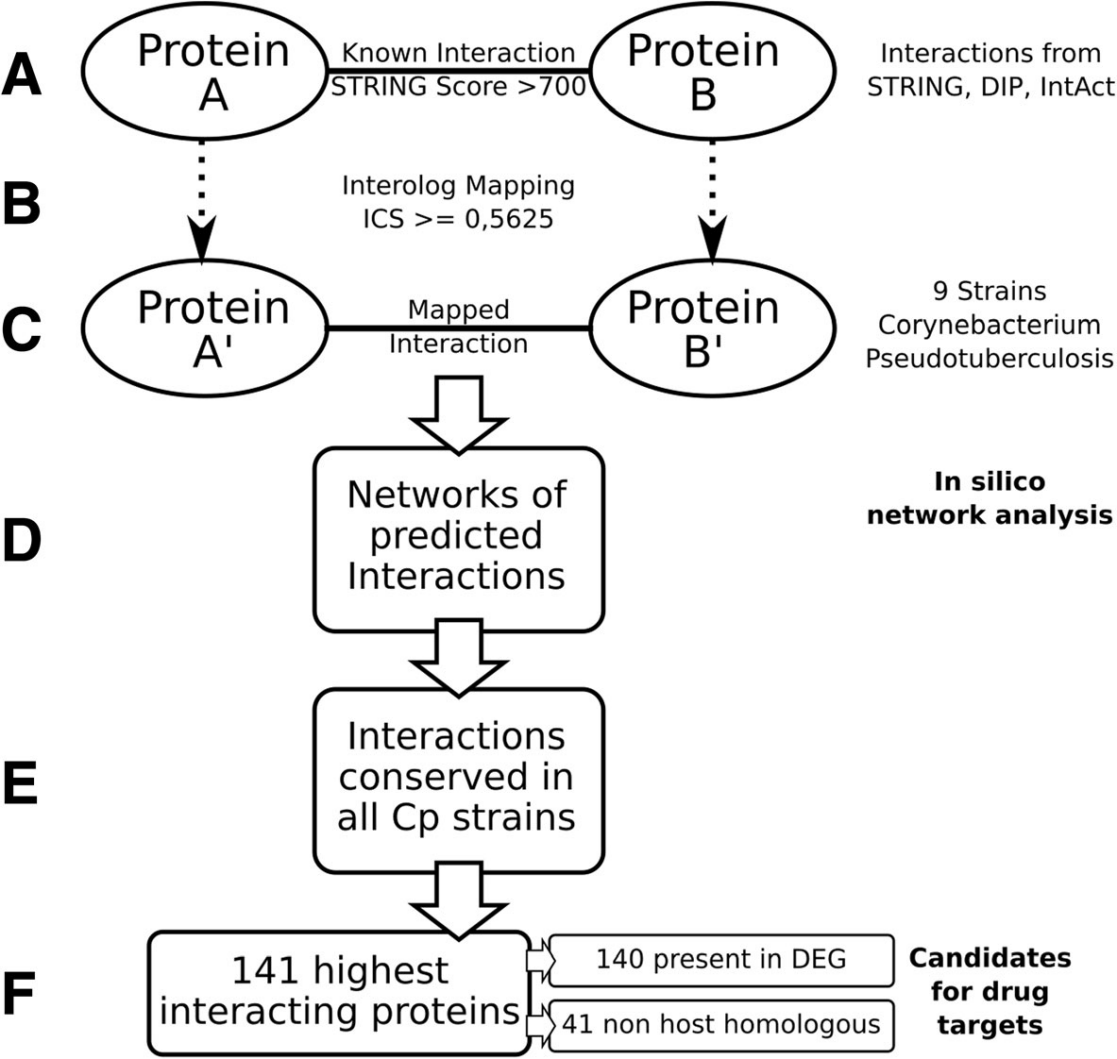
Overview of the workflow utilized in this manuscript. **a** We extracted known interactions from the STRING, DIP, and IntAct databases. **b** For each interaction we searched for conserved counterparts in the nine *Cp* strains and mapped the interaction in case an ICS score of 0.5625 or larger was achieved (**c**). The mapped interactions form the candidate networks for the *Cp* strains (**d**). For the further investigation we only used those interactions which are present in all nine strains (**e**) and extracted the top 15 % proteins with the highest interaction degree (**f**) as they are most likely to be essential proteins. Of the selected 181 proteins were 180 indeed present in the DEG database and furthermore, 41 of those have no homologous counterpart in the host organisms and thus are promising potential drug targets.

We have employed the workflow depicted in Fig. 1 for deriving the candidate list of potential protein-protein interactions. We will give a brief summary of the method before describing the details in the subsequent chapters: We have extracted known regulations of publicly available databases and used those as the basis for our predictions (see subchapter data sources). For each interaction we have searched for conserved counterparts in the *Cp* strains. In case both interaction partners were sufficiently conserved (refer to the subchapter interolog mapping) we assumed the interaction to be a candidate for a potential interaction in the corresponding *Cp* strain. The networks derived with this method then were briefly checked for sanity *in silico*. We continued the analysis by using only potential interactions predicted for all nine *Cp* strains, i.e., the core-interactome. Here, we extracted the 15 % top ranking nodes with respect to the node degree in the networks which represent potentially essential proteins. In fact, we found for all extracted proteins but one an entry of a homologous protein in the DEG database. Further, we compared the potentially essential proteins against the proteome of the host organisms in order to discover essential proteins exclusive to *Cp* which comprise potential drug targets.

### Data sources

The prediction of the PPI networks is based on the protein sequence similarity and the information of already known PPIs. The protein sequences were downloaded from NCBI and the known PPIs were retrieved from three publicly available databases (Table 2). The STRING database [54] is composed of known and predicted PPIs, including direct (physical) and indirect (functional) associations derived mainly from genomic context, high-throughput experiments, co-expression and computational prediction methods. The DIP database [53] contains experimentally determined PPIs that are automatically or manually curated. The IntAct [55] database consists of molecular interaction data derived from literature or direct submissions. The DIP and IntAct databases are curated by the IMEx (International Molecular Exchange) consortium [92]. It is important to note that all databases may contain false-positives, i.e., report an interaction when in fact there is none. This might in particular be true for the largest of the databases, the STRING database. There have been several attempts to filter out false-positives from such databases, e.g., by means of integrating several scores with Bayesian methods [93] or by incorporating inter-species confirmation (i.e., regulations which have been experimentally confirmed in different species) [94]. In this work, the main focus is on the identification of essential proteins (i.e., proteins with a high node degree in the network) thus impact of a limited number of false-positives is reduced. We only employ interactions from the STRING database with a score of above 0.700 (i.e., high-confidence interactions) [95]. Only approximately 10 % of the interactions (around 29 million) are classified in the high or highest confidence categories.

**Table 2:** Overview of the data sources

Data	Proteins	Non redundant interactions	Reference
DIP	23,680	70,630	[53]
STRING	5,214,234	336,561,678	[54]
IntAct	60,846	314,019	[55]

STRING database contains in total of 673,123,356 interactions including duplicate interactions (downloaded in 2014, June). DIP, STRING and IntAct are publicly available and free to use.

### The interolog mapping

In order to transfer the known interactions to the *Cp* strains, we employed the so-called interolog mapping which was already successfully utilized in several other studies [48–50] and essentially corresponds to the “generalized interolog mapping” method as described in Yu et al. [57].

The main assumption of the method is that if two interacting proteins (A and B) have respective orthologous proteins (A’ and B’) in another organism, the orthologous pair also interacts [96]. For the homology detection, we utilized NCBI BLAST [97]. As we are aiming to base our predictions on a wide basis, we employed an as generous E-value cut-off as computationally feasible. We set the E-value parameter to 1e^−5^ for proteins from DIP and IntAct databases and to 1e^−9^ for proteins from the STRING database due to its sheer size. We performed a reciprocal search, meaning each protein was used as subject in one run and as query in the other run (i.e., we search in both directions). For the remainder, we only consider protein pairs that yield a hit in both directions (reciprocal hits). For each reciprocal hit, we compute the prediction score (PS):

1 $$ PS(A)= \min\;\left\{ id\left(A\mathit{\hbox{'}}\to A\right)\cdot cov\left(A\mathit{\hbox{'}}\to A\right), id\left(A\mathit{\hbox{'}}\leftarrow A\right)\cdot cov\left(A\mathit{\hbox{'}}\leftarrow A\right)\right\} $$

where *A* ' represents a protein of *Cp* and *A* the homologous protein of the interaction database, *id*(*A* ' → *A*) the percentage of matching letters in the pair-wise alignment of the sequences and cov(*A* ' → *A*) the length of the alignment compared to the protein length. Finally, we assigned an interaction conservation score (ICS) to each known interaction having homologous proteins in *Cp*:

2 $$ ICS(AB)= min\left(PS(A),PS(B)\right) $$

We considered interactions with an *ICS(AB)* greater than 0.5625 (corresponds to at least 75 % identity and 75 % coverage on average) as conserved. This threshold was derived in a previous study [52] as described in the Background section. When redundant interactions were found (e.g., through a homologous interaction pair of a different organism), the one with highest ICS(AB) was used to compose the PPI network.

### *In silico* PPI network validation

As a first sanity check of our potential PPI network, we aimed to show that the predicted networks show realistic and typical network properties. Therefore, we computed several network statistics and compared them to those of known biological networks. We utilized the Cytoscape [98] plugin NetworkAnalyzer [99] and calculated the shortest path [33, 62, 100], the degree distribution [28], the network topology and the Shapiro-Wilk normality test [59].

Furthermore, we investigated the inherent network structure by performing a cluster analysis. We employed Markov Clustering (MCL) [101] implemented in the Cytoscape plug-in ClusterMaker [102]. We used an inflation parameter of 3.0 for the clustering. To reinforce that these interactions do occur in *Cp*, a literature search was performed to verify the existence of these clusters in phylogenetically close organisms (Additional file 3).

### Essential proteins

In *Saccharomyces cerevisiae* it was shown that the node degree of a protein (i.e., the number of interactions of that protein) is correlated with the lethality of removing that protein from the network [62, 63]. Nodes with a high node degree are called hubs, but a clear definition of what node degree should be regarded as “high” is missing [103]. Nevertheless, identifying nodes with a larger degree is a means for identifying essential proteins [104–106], since the knockout of hub proteins most likely cause a substantial disruption in the interaction network [85]. We decided to classify proteins as essential when they are among the top 15 % proteins with respect to the node degree, a threshold commonly used [103]. Next, to validate essential hub proteins, we searched for homologous sequences stored in DEG [64, 65] (v11.2, updated on July 3, 2015), a database of bacterial essential genes. For the homology detection we again employed BLAST with the following parameters: *e*-value = 1e^−5^, low complexity filter = false and matrix = BLOSUM62. We also aligned the essential proteins of *Cp* against the all proteins of the five host organisms *Ovis aries* (taxid: 9940), *Capra hircus* (taxid: 9925), *Bos Taurus* (taxid: 9913), *Equus caballus* (taixd: 9796), and *Homo sapiens* (taxid: 9606).

## Additional files

Additional file 1: Figure S1. Source organisms of the mapped interactions. (JPG 374 kb)
Additional file 2: Shortest path and degree distribution analysis. Shortest Path analysis of the nine *Corynebacterium pseudotuberculosis* serovar ovis strains and Degree distribution analysis of the nine *C. pseudotuberculosis* serovar ovis strains. (PDF 1004 kb)
Additional file 3: Protein complex analysis. Literature-based description of protein complexes found in the interaction network. (PDF 1723 kb)
Additional file 4: List of 181 essential proteins. The amino acid sequence of hubs proteins was compared against bacterial proteins sequence from Database of Essential Genes (DEG). (TXT 62 kb)
Additional file 5: Figure S2. Homology distribution of Cp essential proteins aligned against hosts. Dark green: proteins homologous to host; Yellow: Proteins with low identity against hosts (identity < 30 %). Dark red: non-host homologous proteins, proteins with low identity and low coverage alignment against hosts (identity x coverage < = 10 %). Dark blue: non-host homologous proteins, proteins with no alignment hits against O. aires and C. hircus. Light blue: non-host homologous proteins, proteins with no alignment hits against the five hosts. The alignment summary is depicted in Additional file 6. (JPG 318 kb)
Additional file 6: Essential protein alignment against host. Blast alignment summary of 181 essential proteins of *Corynebacteria pseudotuberculosis* against the host. (TXT 65 kb)

## Abbreviations

AUC: Area under curve
CLA: Caseous lymphadenitis
CMNR: Corynebacterium Mycobacterium, Nocardia, Rhodococcus
Cp: Corynebacterium pseudotuberculosis
ICS: Interaction conservation score
MCL: Markov clustering
PPI: Protein-protein interactions
PS: Prediction score
