# Supplementary figures and images for: In silico identification of essential proteins in Corynebacterium pseudotuberculosis based on protein-protein interaction networks
Source: BMC Syst Biol. 2016 Nov 4;10:103. doi: 10.1186/s12918-016-0346-4 (PMC5097352; doi:10.1186/s12918-016-0346-4)

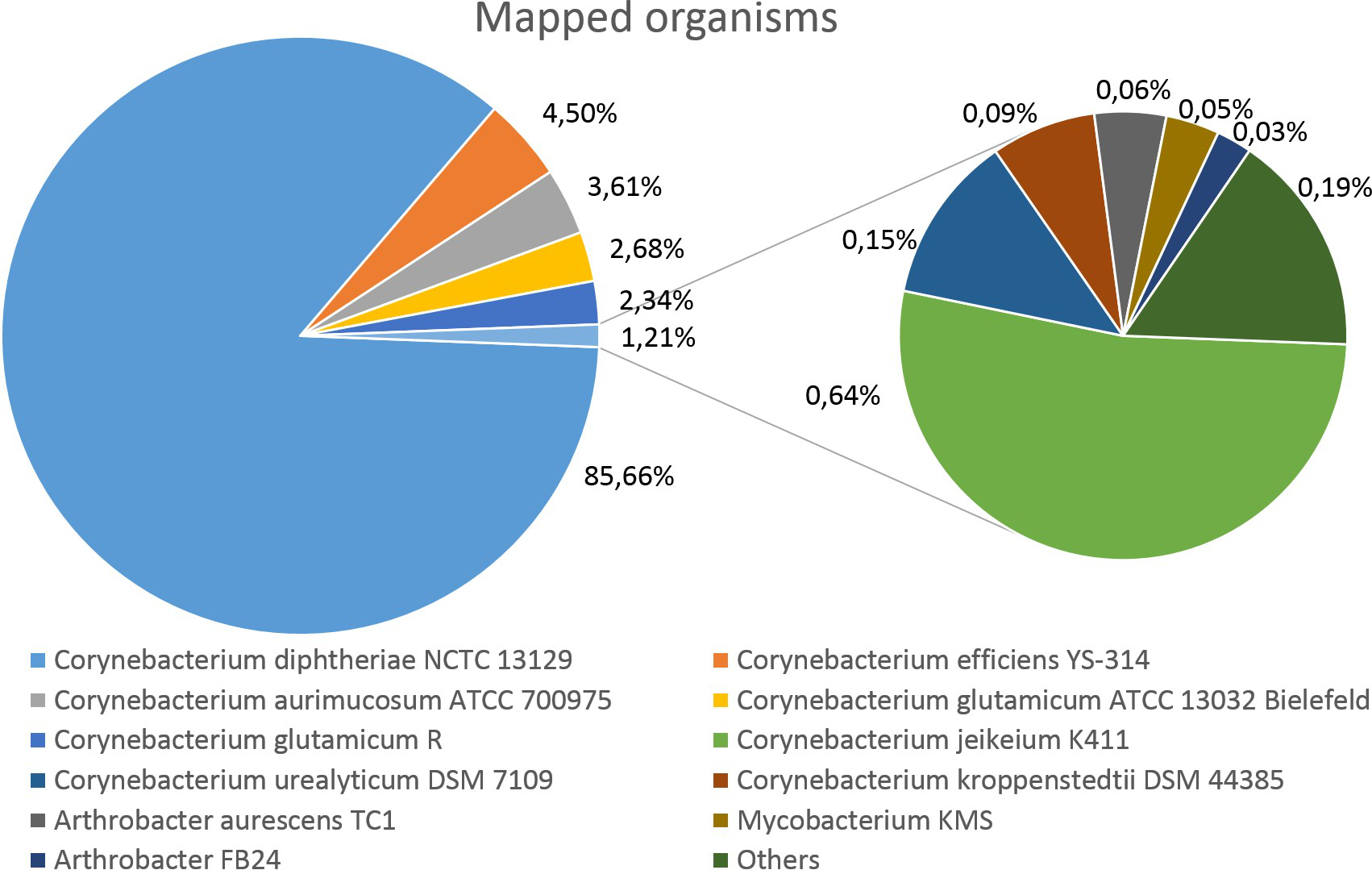

Supplement: Additional file 1: Figure S1. — Source organisms of the mapped interactions. (JPG 374 kb) [file 12918_2016_346_MOESM1_ESM.jpg]

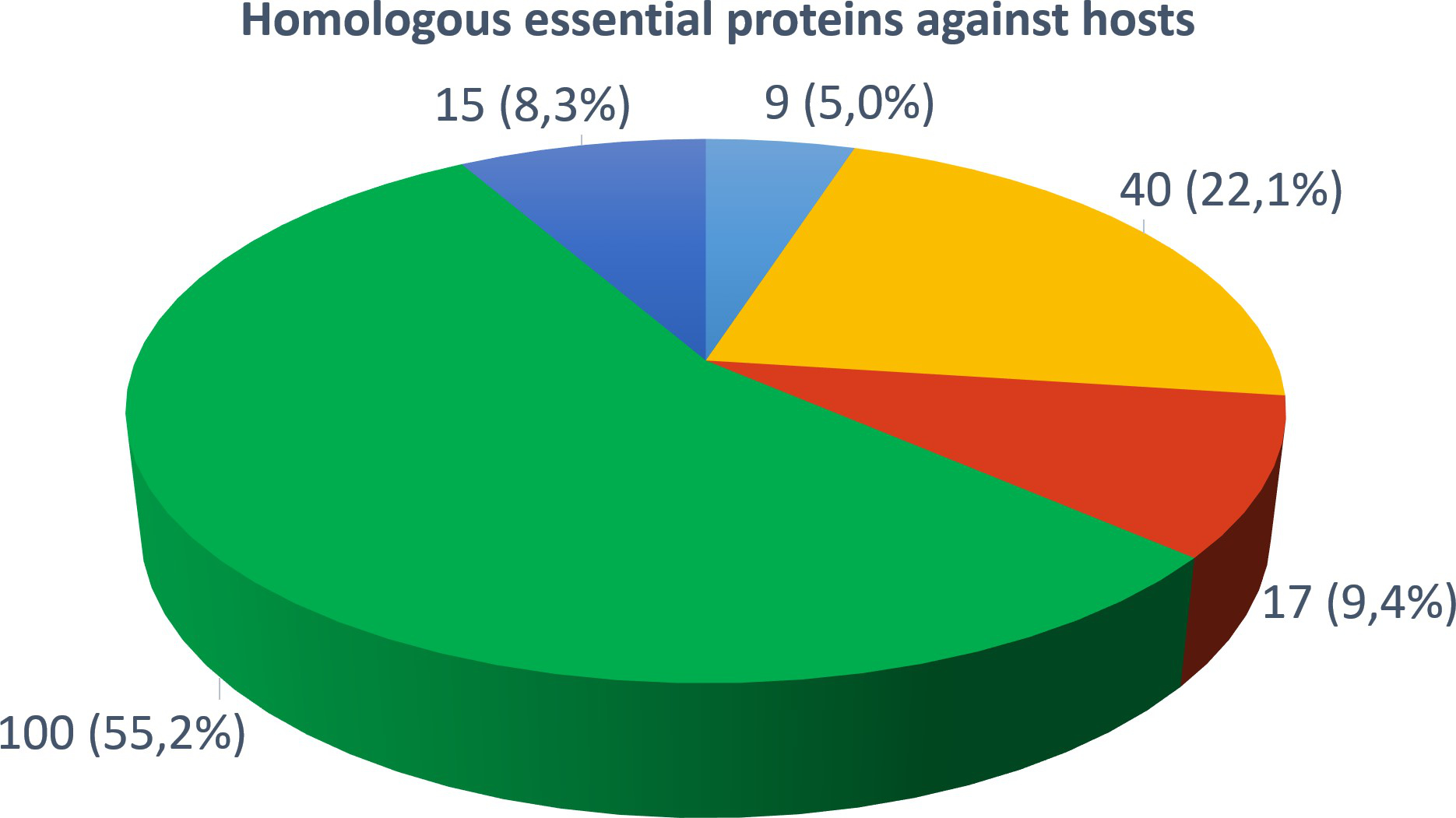

Supplement: Additional file 5: Figure S2. — Homology distribution of Cp essential proteins aligned against hosts. Dark green: proteins homologous to host; Yellow: Proteins with low identity against hosts (identity < 30 %). Dark red: non-host homologous proteins, proteins with low identity and low coverage alignment against hosts (identity x coverage < = 10 %). Dark blue: non-host homologous proteins, proteins with no alignment hits against O. aires and C. hircus. Light blue: non-host homologous proteins, proteins with no alignment hits against the five hosts. The alignment summary is depicted in Additional file 6. (JPG 318 kb) [file 12918_2016_346_MOESM5_ESM.jpg]
