# Supplementary material for: In silico identification of essential proteins in Corynebacterium pseudotuberculosis based on protein-protein interaction networks
Source: BMC Syst Biol. 2016 Nov 4;10:103. doi: 10.1186/s12918-016-0346-4 (PMC5097352; doi:10.1186/s12918-016-0346-4)
Supplement: Additional file 2: — Shortest path and degree distribution analysis. Shortest Path analysis of the nine Corynebacterium pseudotuberculosis serovar ovis strains and Degree distribution analysis of the nine C. pseudotuberculosis serovar ovis strains. (PDF 1004 kb) [file 12918_2016_346_MOESM2_ESM.pdf]

Additional File 2

Shortest path and degree distribution analysis. Shortest Path analysis of the nine *Corynebacterium pseudotuberculosis* serovar ovis strains (Figure 1-9). Degree distribution analysis of the nine *C. pseudotuberculosis* serovar ovis strains. The red line indicate the perfect power-law distribution (Figure 10-18).

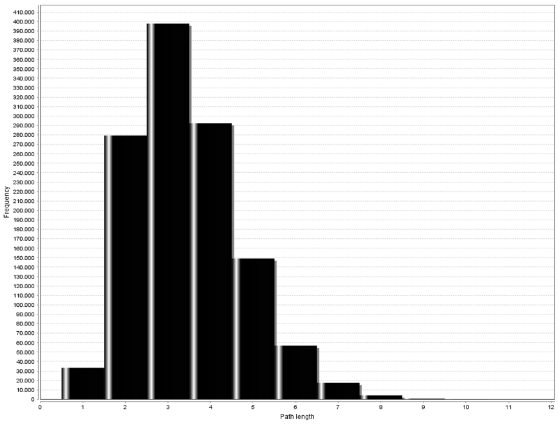

Figure 1 - Cp1002 Shortest Path analysis

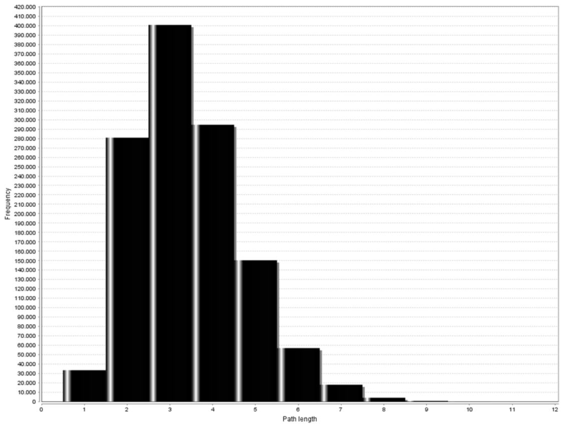

Figure 2 - Cp267 Shortest Path analysis

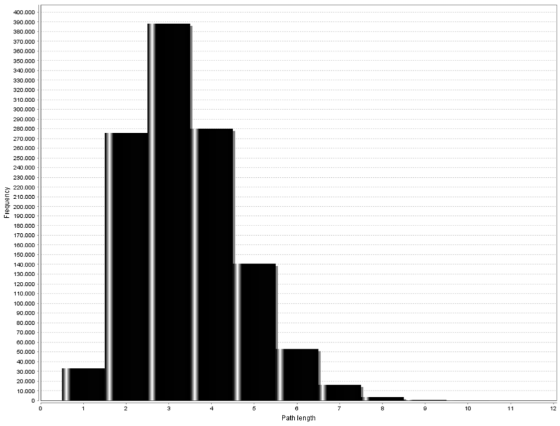

Figure 3 - Cp3995 Shortest Path analysis

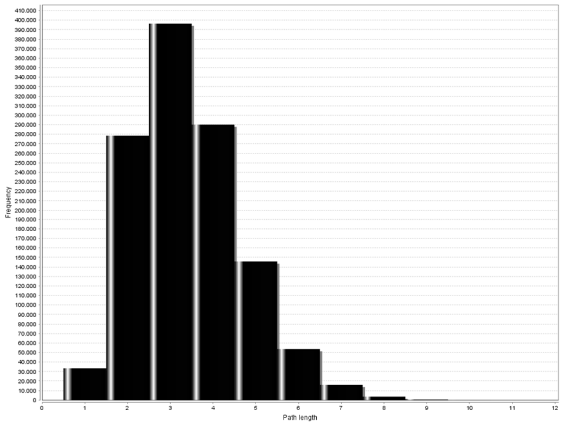

Figure 4 - Cp4202 Shortest Path analysis

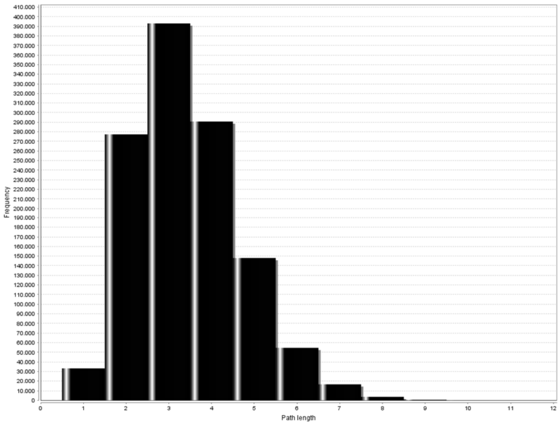

Figure 5 - CpC231 Shortest Path analysis

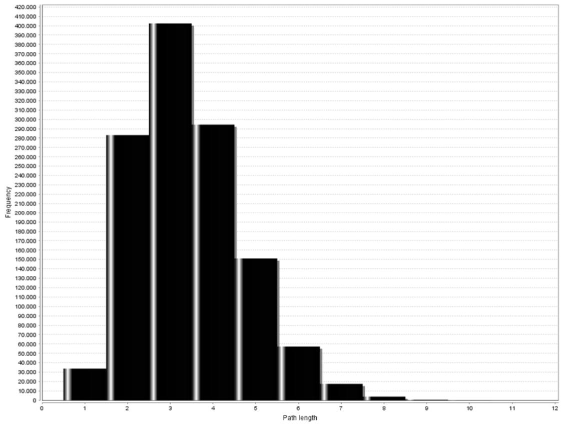

Figure 6 - Cpfrc Shortest Path analysis

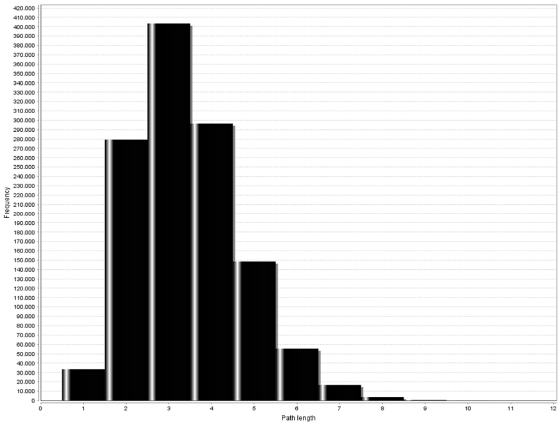

Figure 7 - CpI19 Shortest Path analysis

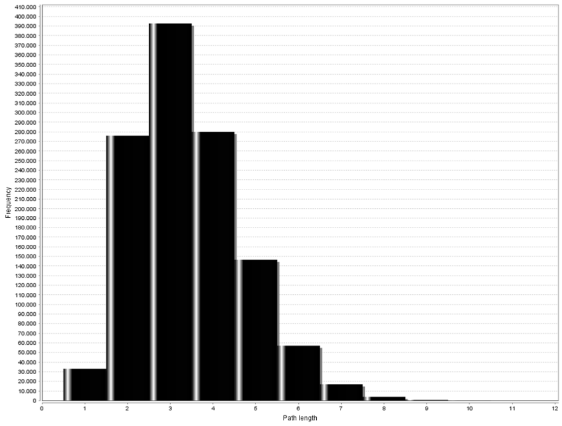

Figure 8 - CpP54B96 Shortest Path analysis

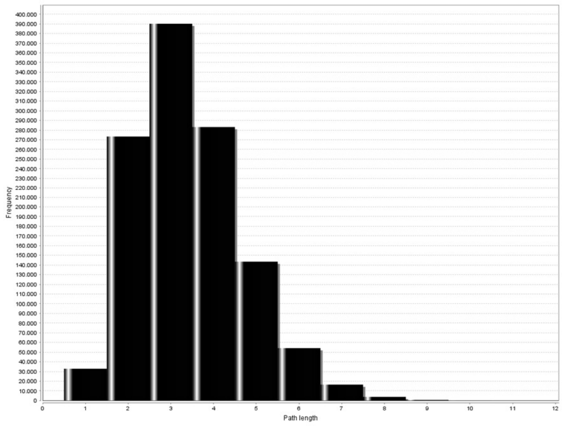

Figure 9 - CpPAT10 Shortest Path analysis

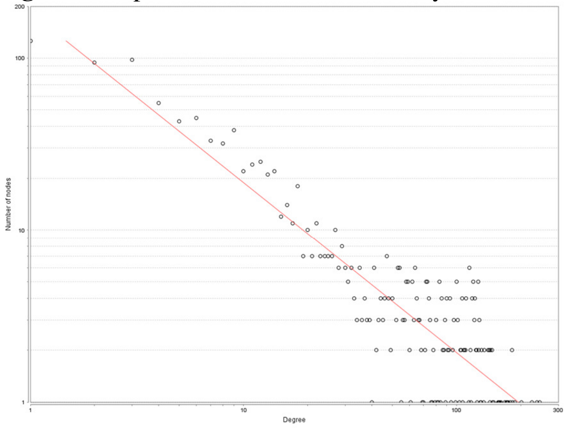

Figure 10 - CpPAT10 Degree distribution analysis. Clustering coefficient = 0.407, Correlation = 0.938, R-Squared = 0.790, Shapiro-Wilk test = p-value < 2.2e-16.

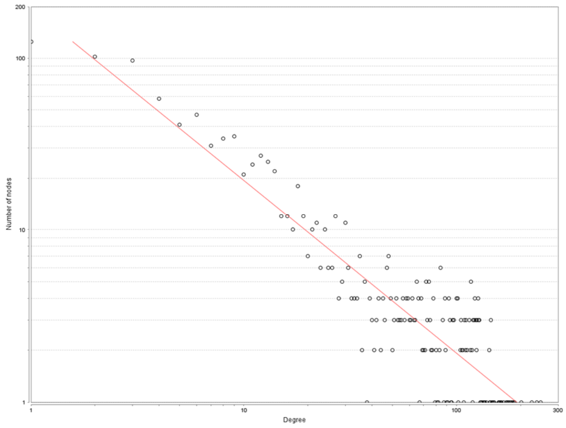

Figure 11 - Cp1002 Degree distribution analysis. Clustering coefficient = 0.408, Correlation = 0.933, R-Squared = 0.822, Shapiro-Wilk test = p-value < 2.2e-16.

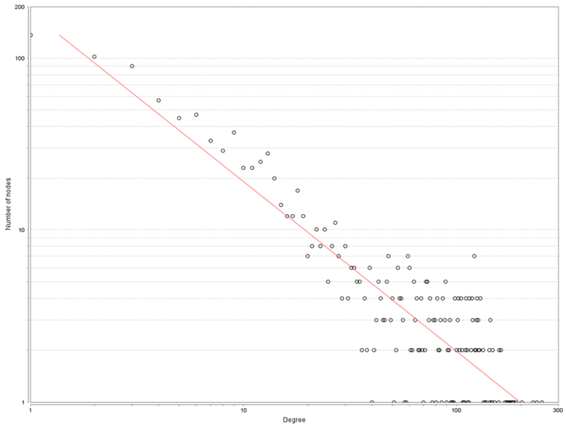

Figure 12 - Cp267 Degree distribution analysis. Clustering coefficient = 0.402, Correlation = 0.953, R-Squared = 0.785, Shapiro-Wilk test = p-value < 2.2e-16.

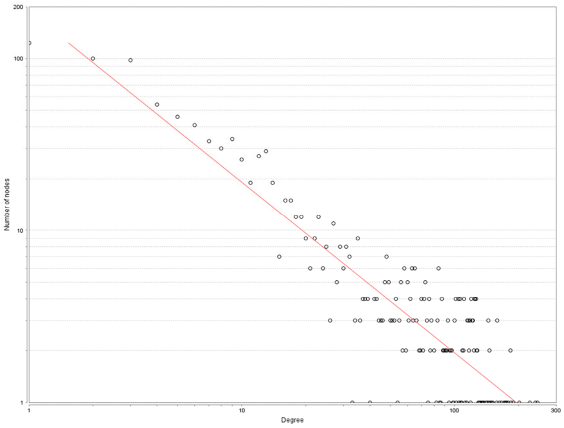

Figure 13 - Cp3995 Degree distribution analysis. Clustering coefficient = 0.410, Correlation = 0.933, R-Squared = 0.798, Shapiro-Wilk test = p-value < 2.2e-16.

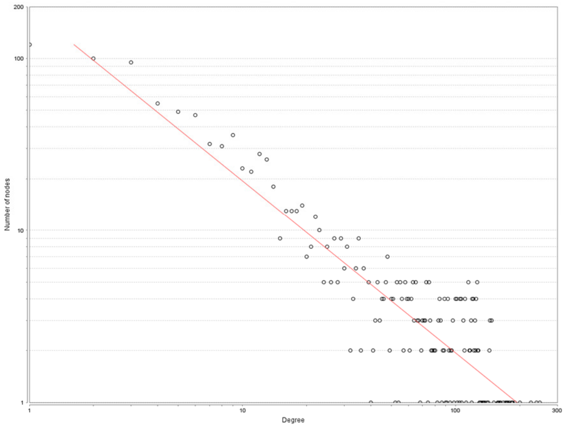

Figure 14 - Cp4202 Degree distribution analysis. Clustering coefficient = 0.410, Correlation = 0.928, R-Squared = 0.799, Shapiro-Wilk test = p-value < 2.2e-16.

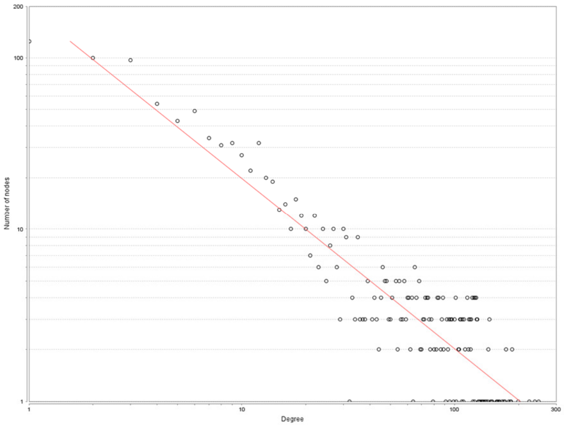

Figure 15 - CpC231 Degree distribution analysis. Clustering coefficient = 0.407, Correlation = 0.936, R-Squared = 0.825, Shapiro-Wilk test = p-value < 2.2e-16.

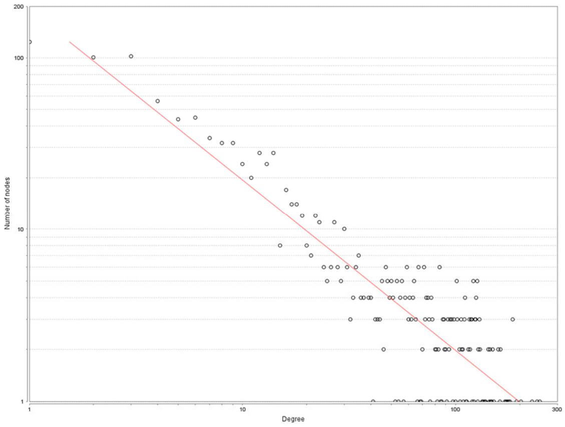

Figure 16 - Cpfrc Degree distribution analysis. Clustering coefficient = 0.408, Correlation = 0.930, R-Squared = 0.786, Shapiro-Wilk test = p-value < 2.2e-16.

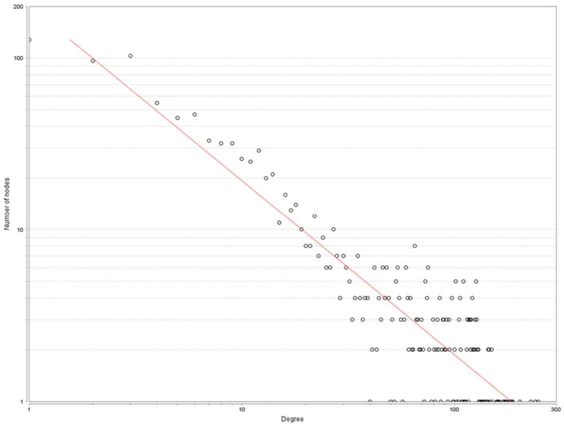

Figure 17 - CpI19 Degree distribution analysis. Clustering coefficient = 0.403, Correlation = 0.932, R-Squared = 0.813, Shapiro-Wilk test = p-value < 2.2e-16.

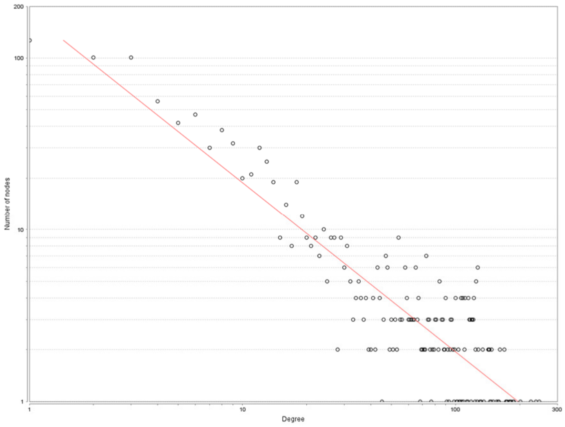

Figure 18 - CpP54B96 Degree distribution analysis. Clustering coefficient = 0.404, Correlation = 0.935, R-Squared = 0.800, Shapiro-Wilk test = p-value < 2.2e-16.
