# Supplementary material for: In silico identification of essential proteins in Corynebacterium pseudotuberculosis based on protein-protein interaction networks
Source: BMC Syst Biol. 2016 Nov 4;10:103. doi: 10.1186/s12918-016-0346-4 (PMC5097352; doi:10.1186/s12918-016-0346-4)
Supplement: Additional file 3: — Protein complex analysis. Literature-based description of protein complexes found in the interaction network. (PDF 1723 kb) [file 12918_2016_346_MOESM3_ESM.pdf]

Additional File 3

Protein complex analysis

Protein complexes are either formed by groups of identical proteins (homomers) or different proteins (heteromers), and their organization is important in performing specific biological activities in a biological process [1]. Such complexes are subject to evolutionary selection [2] and they have a degree of conservation between species. In an interaction network, complexes may be identified by investigating densely connected proteins, the so-called clusters [3]. To identify the clusters in the predicted networks, we used the Markov Cluster Algorithm (MCL) with inflation parameter set to 3.0 [4]. We used the implementation of the Cytoscape [5] plug-in ClusterMaker [3]. In addition, to validate the interaction networks, a literature search was performed to verify the existence of similar clusters in other organisms, in the form of operons or metabolic pathways.

Network Figures

All provided network pictures follow the same color coding to visualize further information. The node size (from small to large) and color (in a range from yellow to light green to dark green) encodes the node degree. The border size (from small to large) and color (in a range of white, pink and dark red) represent the "Betweenness Centrality" property. The edge color (from red to yellow to green) represents the confidence score of the original interaction assigned by the database. The edge width, from thinner to wide, represents the interaction score pair (ISP).

Ribosomal and RNA polymerase cluster

This complex comprises of protein-protein interactions (PPI) occurring during the translational process of ribosomes (ribosomal RNAs + protein) in

C. pseudotuberculosis (*Cp*). This complex is formed by 53 ribosomal proteins (RP) and 4 of the 5 proteins forming the RNA polymerases (RNAP). All proteins are conserved in all *C. pseudotuberculosis* biovar *ovis* strains. The RPs in this complex are encoded by 23 *rpl* genes (*rplBICEMKAQSDNLTFPOVJRWUXY*), 10 *rpm* genes (*rpmAEHBDCGIFJ*) and 20 *rps* genes (*rpsLBKIDEOJGCMHARSPNFQT*) [6]. The RNAP proteins are encoded by genes *rpoA*, *rpoB*, *rpoC* and *rpoZ* [7, 8] (Figure 1). The complex contains operons containing genes encoding ribosomal proteins and genes encoding proteins that form the subunits of RNAP: for instance, the rplKAJL-rpoBC operon encoding the proteins of a large subunit of a ribosome and also the β and β' subunits of RNAP [7]. As in all prokaryotes, the transcriptional and translational systems are coupled and synchronized in space and time rendering RNAP and RP relevant for understanding the dependence between these two processes [9]. *Escherichia coli* was the first organism having the ribosomal component (rRNA + proteins) elucidated [10], and hence is being widely used as a model for studies of ribosomal gene clusters in bacteria due to the similarity in the formation and organization of these clusters. In *C. glutamicum* and *C. diphtheriae*, eleven gene clusters encoding 42 ribosomal proteins have been described and when comparing with the *E. coli* gene clusters, seven of the discovered *Cp* clusters are organized in the same way and four cluster have highly similar proteins [11].

Several recent studies target the relationship between the ribosomal machinery and RNAP. In one study, it was observed that the complex formed by the proteins encoded by the genes *nusG-rpsJ*, bind RNAP to the 30S subunit of the prokaryotic ribosome [12]. In another study, the gene that encodes the S1 protein also binds to RNAP and stimulates transcriptional activity [13]; these interactions are also observed in the predicted networks presented here.

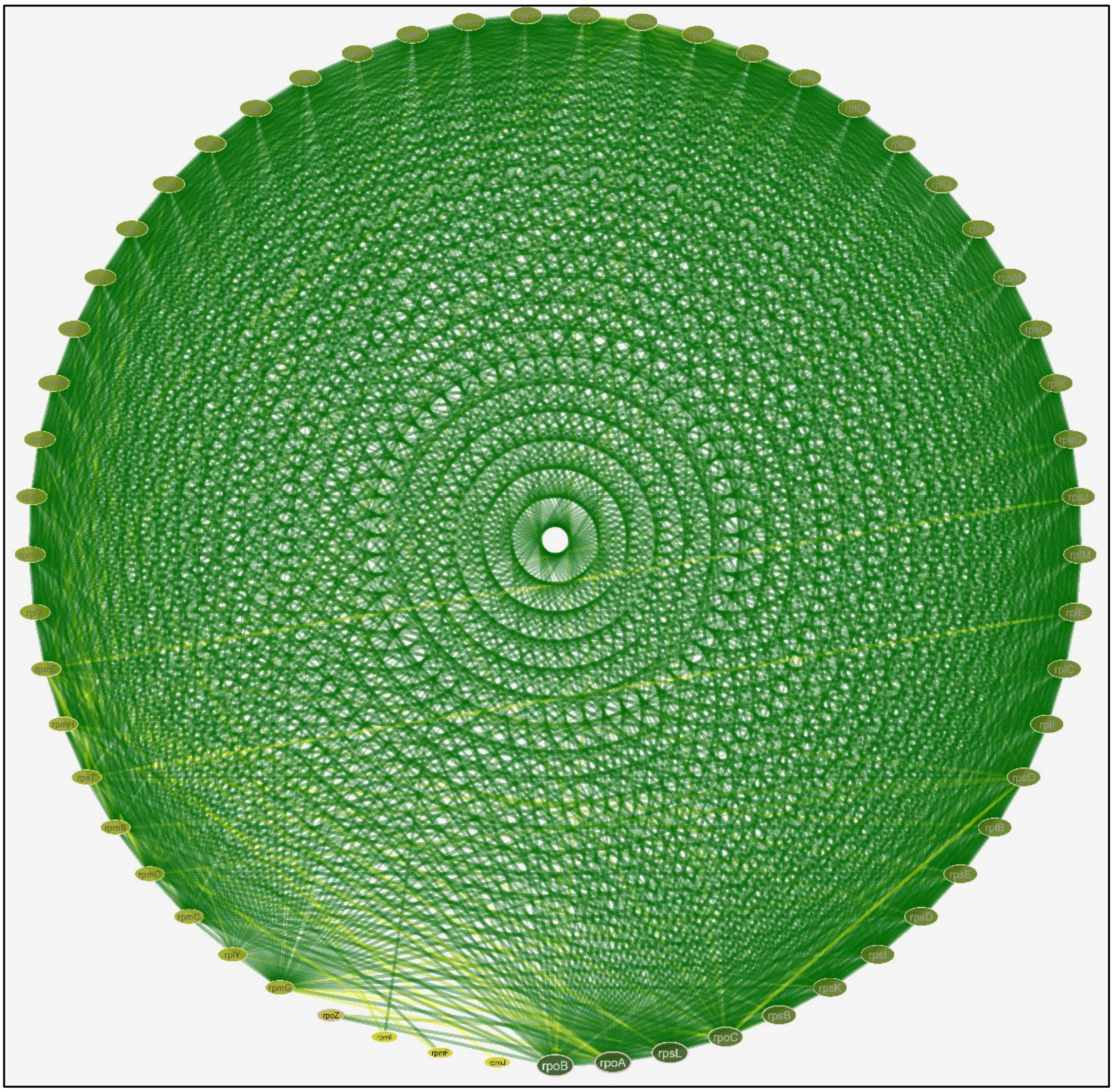

Figure 1. Network formed by the interaction of RNA polymerase and ribosomal proteins, represented by their encoding gene.

One further important observation confirms the biological bias of the predicted interaction network: a multitude of interactions of proteins encoded by genes *rpoB*, *rpoC*, and *rpoA* with RP and in contrast, no interactions of the protein encoded by the gene *rpoZ* with RP. This can be justified by the fact that *rpoZ* is a sigma factor responsible for recognizing the binding site. After the protein beta subunits (β-encoded by *rpoB* gene), beta' (β'- encoded by gene *rpoC*) and alpha (α-encoded by *rpoA* gene) form the RNAP, *rpoZ* disconnects from the binding site. The network analysis can help us also select molecular targets for possible drug action. The proteins encoded by *rpoA* gene, *rpoB* and

rpoC are highly connected to proteins of RP. Thus, they can potentially serve as candidate targets for drug development. An example of a successful similar drug is the RNAP β subunit inhibition (encoded by the *rpoB* gene) by antibiotic Rifampicin. There are also antibiotics like tetracycline, paromomycin, spectinomycin and streptomycin that exert their inhibitory activity on some proteins in the ribosomal 30S complex [14].

Oligopeptide transport system cluster

The Opp transporters belonging to the ABC transporters family (ATP-binding cassette) were identified and characterized in several bacterial species, both in gram-positive and gram-negative [15, 16]. This system consists of five protein subunits: OppA, responsible for the peptides capture of extracytoplasmic means; OppB and OppC form the transmembrane channel through which the oligonucleotides will be transported to the intracellular environment; OppD and OppF are located in the bacterial cytoplasm and are responsible for the hydrolysis of ATP molecules generating power for the process of internalizing peptides [16]. From a genetic point of view, the genes encoding these subunits are organized as an operon *oppABCDF* [17] (Figure 2). In bacteria, the main function of *Opp* is the peptide acquisition utilized as carbon and nitrogen source. In *E. coli,* it was demonstrated that this system is associated with the residues internalization of various amino acid types [18]. A study of *Lactococcus lactis* has shown that the presence of a functional peptide transport system is required for the growth of bacteria in milk [19]. According to the generated interaction network, the Opp system is directly linked to the protein dihydrodipicolinate synthase (nanL) participating in L-lysine biosynthesis suggesting that this system may be associated with amino acids biosynthesis [20]. To date, no study was conducted to demonstrate the role of the Opp system in the transport of essential and nonessential amino acids in *C. pseudotuberculosis*. However, was shown that the *Opp* system contribute to the adhesion

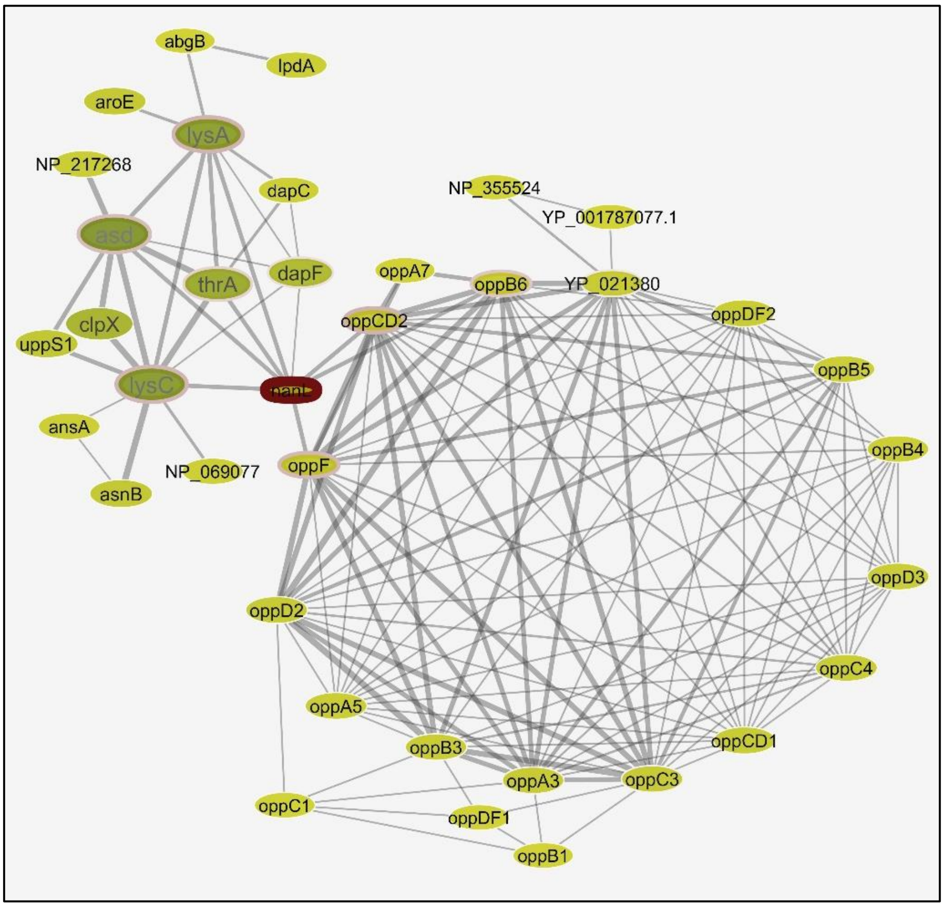

Figure 2. Network formed by the interaction of Opp proteins, represented by their encoding genes

process of this pathogen [21]. In *Moraxella catarrhalis*, it was demonstrated that the Opp system is also involved in the acquisition of arginine and contributes to the fitness and persistence of the pathogen in the respiratory tract [22]. These studies demonstrate the versatility of the Opp system in pathogenic bacteria.

Cobalamin biosynthesis cluster

Cobalamin (CBL - Vitamin B_{12}) is synthesized by a number of Archaea and Bacteria [23, 24]. However, the prosthetic group CBL is essential for the enzymatic activity of several enzymes in all three biological domains [25]. In Bacteria and Archaea, the functional dependency is present in the CBL methionine synthase, ribonucleotide reductase, glutamate, methylmalonyl-coA mutases, ethanolamine ammonia lyase, etc. [26]. The

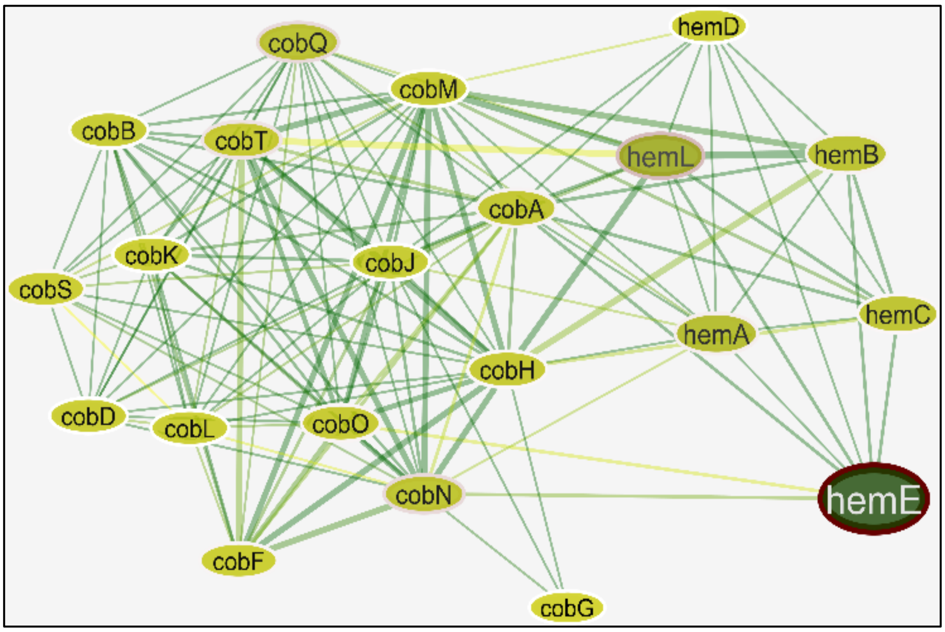

Figure 3. Network formed by the interaction of Cob proteins, represented by their encoding genes

biosynthesis pathways of CBL cofactors, chlorophyll and haem begin with the compound 5-aminolevulinic acid (ALA). This, through some enzymatic steps, is converted into Uroporphyrinogen III, the last common intermediate compound for tetrapyrrolic products [25, 27, 28]. In the predicted PPI network for *C. pseudotuberculosis* we can observe in the CBL complex the presence of several holoenzymes (*HemABCDEL*) interconnected with the holoenzymes (*CobABDFGHJKLMNOQST*) (Figure 3). This suggests a co-evolutionary dependence between these two systems. For cobalamin production, multiple steps and structural rearrangement of transmethylation are required [26]. In *C. pseudotuberculosis*, these reactions are catalyzed by 15 cob genes, with most of them being in the main cob operon, while the remaining genes (*cobA, cobB, cobC and cobD*) are not present in the main operon. This fact may indicate the contribution of these genes to external assimilation of vitamin B_{12} precursors or secondary processes of de novo biosynthesis, as identified in *Pseudomonas denitrificans* [23]. The cbi gene cluster (cobinamide), responsible for CBL biosynthesis by an anaerobic pathway [29], is absent

in the network; so we can postulate that *C. pseudotuberculosis* might solely use the aerobic pathway as an alternative to produce CBL [26].

Iron uptake and intracellular regulation cluster

This complex is responsible for the capture process and intracellular regulation of iron (Fe). Fe is an essential cofactor for diverse enzymatic activities that work in different metabolic processes (e.g., DNA replication, ATP synthesis, DNA repair and respiration etc.) in all eukaryotic organisms and various prokaryotes [30-32]. In pathogenic bacteria such as *C. pseudotuberculosis*, the Fe^{+} ions acquisition system, contributes to the survival and virulence of the microorganism [33, 34]. A single bacterium can have multiple Fe acquisition systems. This feature is used as a strategy to acquire Fe from different sources and in low availability of this cofactor [35]. Thus, the complex represents these multiple systems and consists of 22 proteins encoded by genes *fagABCD, ciuABCD, fecCDE (CD), hmuUVTO, htaA, pstA, fhuD, fpeC1, hemE* and *dtxR* (**Figure 4**).

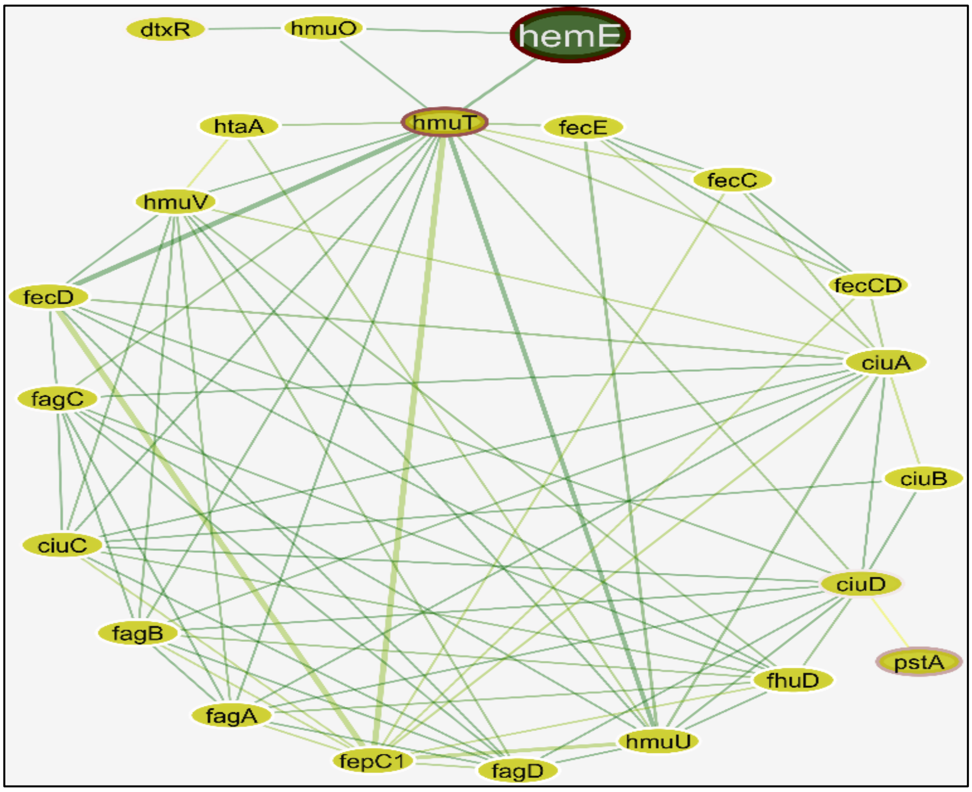

Figure 4. Network formed by the interaction of Iron uptake proteins, represented by their encoding genes.

During the infection process, *C. pseudotuberculosis* is able to survive and multiply within macrophages and hence escape from the host immune system response [32]. The use of distinct or multiple siderophores (SIDS) [36] synthesized by *C. pseudotuberculosis* or captured from the external environment [31] is a crucial factor for this ability. In *C. pseudotuberculosis*, the SIDS are synthesized by genes *fagD* [37] (represented in the network) and *ciuE* [32] (not present in the network). The reason for this might be that these SIDS compete for the Iron ion (Fe^{+}) with iron transporters used by the macrophage [31]. Another source of Fe^{+} originates from the transfer of the prosthetic group heme-Fe to the inside of *C. pseudotuberculosis* through *hmuT* receiver whose interactions between *hmuT* and *hemE* can be seen in the network. Once heme-Fe is transported inside, it suffers a degradation process, releasing Fe^{+}. In this process of degradation, hmuO operates in the cleavage of the tetrapyrrole ring of the group Heme-Fe [37]. Additionally, the protein Cell-surface hemin receptor (htaA) exclusively interacts with proteins encoded by the *hmuTUV* genes, responsible for hemin binding and transport. These interactions agree with the literature evidence on *C. diphtheriae* [38]. These observations suggest that (a) the interaction network is consistent and (b) that *C. pseudotuberculosis* can use the same strategy for iron acquisition as *C. diphtheriae*. In the network, there are also other systems for capturing iron, such as: Fag, Fec and Ciu proteins, as part of *C. pseudotuberculosis* strategy to acquire Fe^{+}. The successful 'Trojan Horse' strategy to combat resistant bacteria uses the iron uptake system to enter and kill the cell. The idea is based on the synthesis of the siderophore-drug complex, thus making the iron acquisition pathways through siderophore as potential targets for drug delivery [39]. Recently, a detailed review about iron acquisition strategies of gram-positive pathogens was published where the same cluster proteins are identified, confirming the integrity of the predicted interaction network. Iron, being an important substance for the survival of gram-positive bacteria,

and the mechanisms of iron acquisition, transportation and processing naturally become important areas of study enabling the development of new strategies to combat these organisms [40].

Cell division and peptidoglycan biosynthesis

In various bacteria exist a coupling and fine coordination between the processes related to cell division (cytokinesis), the formation of the peptidoglycan layer that makes up the cell walls, and DNA replication and segregation systems [41, 42]. We identified 36 proteins of *C. pseudotuberculosis* involved in this process and depict their predicted interactions in **Figure 5**. The FtsZIWHYXE proteins are reported to be involved in cell division [41, 43] and the MurAFDEGIBC proteins in the biosynthesis of peptidoglycans [44]. In the cytokinesis process, the FtsZ protein plays a central role in the formation of the cytoplasmic membrane ring constriction and in the anchoring and recruitment of another protein set related to the cell division process [41, 43]. In the network, the FtsZ protein is highly connected making it a central element of the recruitment activity and anchoring. As FtsZ is the main component of the cell division process, there is a need to maintain a harmony with the enzymes relating to the new cell wall synthesis [45]. In the *C. pseudotuberculosis* network, these enzymes are mainly represented by MurABCDEFGI and mraY proteins, related to the synthesis of new multilayer peptidoglycans cell walls [46]. Indeed, our predicted network also shows a possible harmony between the components responsible for the peptidoglycan biosynthesis and the FtsZ protein. It is worth noting the role of the FtsW protein in nascent peptidoglycan transport to the outside of the plasma membrane. In the network, we observe the presence of the proteins encoded by the genes *parA, parB* and *smc* which are related to the chromosome partitioning process; *soj* is associated with ATPase activity and *scpA* related to the condensation process and the bacterial chromosome segregation during cytokinesis.

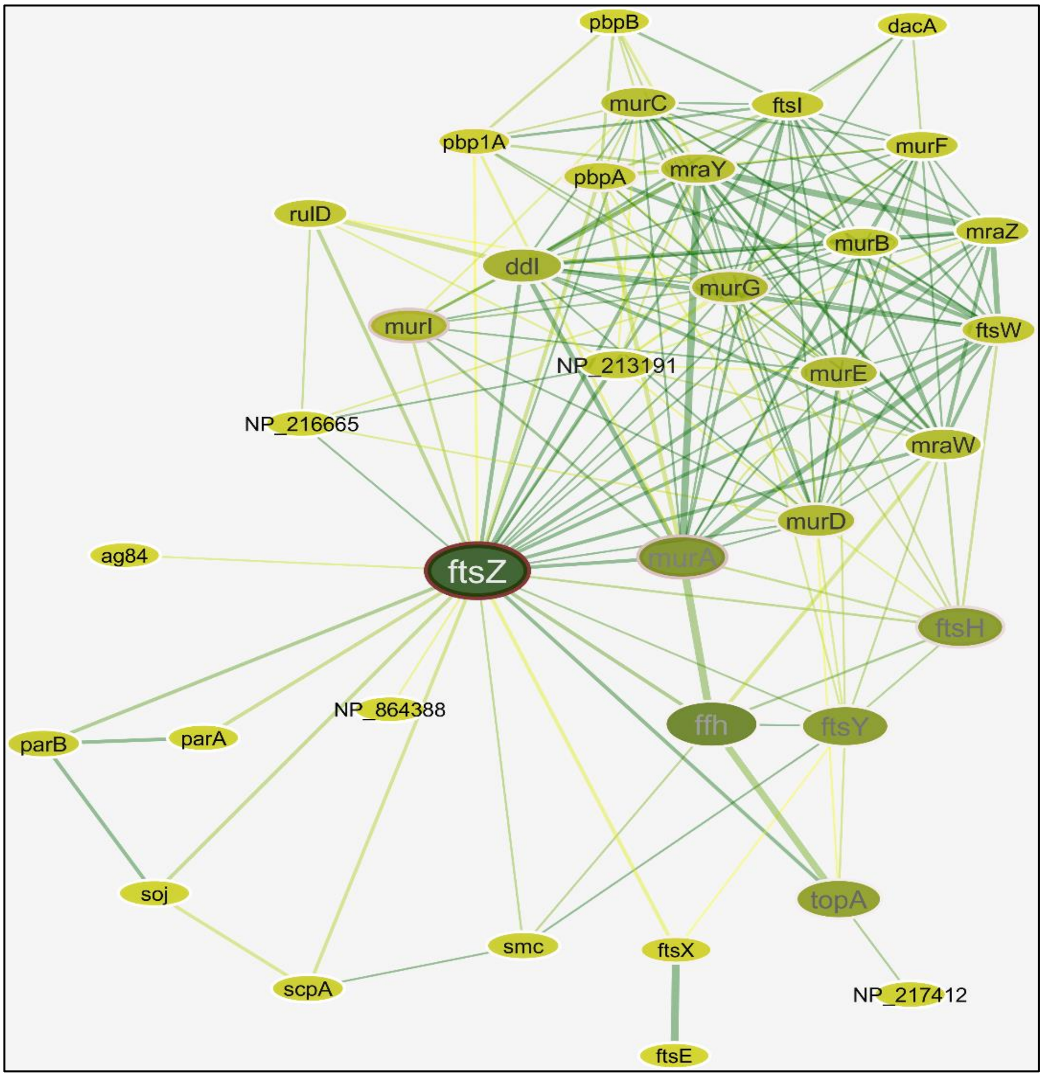

Figure 5. Network formed by the interaction of proteins involved in cell division and peptidoglycan biosynthesis, both represented by their encoding genes.

These proteins mainly interact with FtsZ, showing that FtsZ serves as a support for these proteins to perform their activities accordingly. Complementary approaches using PPI networks can be of great value to overcome the challenge of battling the increasing number of resistant pathogenic bacteria. Thus, the organization and the connection between the network elements can help to identify and to select new molecular targets for the development of more effective therapies. Currently, there are several compounds being synthesized and directed to act in the inhibition of peptidoglycan synthesis and in cell division steps [47]. For example, compounds such as fosfomycin (phosphomycin),

4-thiazolidinone and phosphinic acid derivatives act as inhibitors of MurA, MurB and MurCDEF respectively [44]. In this case, the bacterium dies by failing to form the peptidoglycan layers. Inhibitors directed to block the beginning of cell division by preventing the formation of the constriction ring have been explored and tested. For instance, the sanguinarine inhibitor is not specific to FtsZ although it shows inhibitory activity [47]. Therefore, further studies are needed to find more efficient inhibitors and most promising targets against various bacteria, especially against *C. pseudotuberculosis*.

In general, the clusters whose proteins are described in the literature (although in other organisms), demonstrate the consistency of our predicted interaction network, indicating that the interactions may truly occur in Cp *ovis*. An example are the proteins of the iron acquisition cluster which were also identified in a recent review [40]. It is common that some proteins occur in several clusters, possibly exerting different functions in each cluster. For instance, this is the case of Iron uptake, Cobalamin biosynthesis and Heme clusters, whose cooperation was characterized and described in other organisms [33].

Likewise, clusters or interactions not previously described or only poorly characterized in the literature might lead to novel and relevant insights about Cp *ovis*. From the cluster analysis, we conclude the following: (a) some proteins, operons and interaction participants in the clusters are well described in the literature for other gram-positive organisms, indicating that the predicted interaction networks are biologically feasible for Cp *ovis* and (b) although some proteins and operons are well described in the literature, in some cases, the interactions between these elements are not. Hence, the interaction network has the potential to contribute additional information leading to a better understanding of Cp *ovis*. The lack of information in the literature especially for non-model organisms renders such a predicted PPI network a powerful tool.

BIBLIOGRAPHY

1. Dai Q-G, Guo M-Z, Liu X-Y, Teng Z-X, Wang C-Y: **CPL: Detecting Protein Complexes by Propagating Labels on Protein-Protein Interaction Network**. *Journal of Computer Science and Technology* 2014, **29**(6):1083-1093.
2. Marsh JA, Hernández H, Hall Z, Ahnert SE, Perica T, Robinson CV, Teichmann SA: **Protein complexes are under evolutionary selection to assemble via ordered pathways**. *Cell* 2013, **153**(2):461-470.
3. Morris JH, Apeltsin L, Newman AM, Baumbach J, Wittkop T, Su G, Bader GD, Ferrin TE: **clusterMaker: a multi-algorithm clustering plugin for Cytoscape**. *BMC bioinformatics* 2011, **12**(1):436.
4. Van Dongen S: **A cluster algorithm for graphs**. *Report-Information systems* 2000(10):1-40.
5. Shannon P, Markiel A, Ozier O, Baliga NS, Wang JT, Ramage D, Amin N, Schwikowski B, Ideker T: **Cytoscape: a software environment for integrated models of biomolecular interaction networks**. *Genome research* 2003, **13**(11):2498-2504.
6. Haddadin FaT, Harcum SW: **Transcriptome profiles for high-cell-density recombinant and wild-type Escherichia coli**. *Biotechnology and bioengineering* 2005, **90**(2):127-153.
7. Teixeira D, Eveillard S, Sirand-Pugnet P, Wulff A, Saillard C, Ayres A, Bové J: **The tufB–secE–nusG–rplKAJL–rpoB gene cluster of the liberibacters: sequence comparisons, phylogeny and speciation**. *International Journal of Systematic and Evolutionary Microbiology* 2008, **58**(6):1414-1421.
8. Coenye T, Vandamme P: **Organisation of the S10, spc and alpha ribosomal protein gene clusters in prokaryotic genomes**. *FEMS microbiology letters* 2005, **242**(1):117-126.
9. McGary K, Nudler E: **RNA polymerase and the ribosome: the close relationship**. *Current opinion in microbiology* 2013, **16**(2):112-117.
10. Stelzl U, Connell S, Nierhaus KH, Wittmann-Liebold B: **Ribosomal proteins: role in ribosomal functions**. *eLS* 2001.
11. Martín JF, Barreiro C, González-Lavado E, Barriuso M: **Ribosomal RNA and ribosomal proteins in corynebacteria**. *J Biotechnol* 2003, **104**:41-53.
12. Castro-Roa D, Zenkin N: **In vitro experimental system for analysis of transcription–translation coupling**. *Nucleic acids research* 2012, **40**(6):e45-e45.
13. Sukhodolets MV, Garges S: **Interaction of Escherichia coli RNA polymerase with the ribosomal protein S1 and the Sm-like ATPase Hfq**. *Biochemistry* 2003, **42**(26):8022-8034.
14. Adékambi T, Drancourt M, Raoult D: **The rpoB gene as a tool for clinical microbiologists**. *Trends in microbiology* 2009, **17**(1):37-45.
15. Monnet V: **Bacterial oligopeptide-binding proteins**. *Cellular and Molecular Life Sciences CMLS* 2003, **60**(10):2100-2114.
16. Braibant M, Gilot P: **The ATP binding cassette (ABC) transport systems of Mycobacterium tuberculosis**. *FEMS microbiology reviews* 2000, **24**(4):449-467.
17. Hiron A, Borezée-Durant E, Piard J-C, Juillard V: **Only one of four oligopeptide transport systems mediates nitrogen nutrition in Staphylococcus aureus**. *Journal of bacteriology* 2007, **189**(14):5119-5129.

18. Naider F, Becker JM: **Multiplicity of oligopeptide transport systems in Escherichia coli**. *Journal of bacteriology* 1975, **122**(3):1208-1215.
19. Smid EJ, Plapp R, Konings W: **Peptide uptake is essential for growth of Lactococcus lactis on the milk protein casein**. *Journal of bacteriology* 1989, **171**(11):6135-6140.
20. Kuroda M, Kuroda H, Oshima T, Takeuchi F, Mori H, Hiramatsu K: **Two-component system VraSR positively modulates the regulation of cell-wall biosynthesis pathway in Staphylococcus aureus**. *Molecular microbiology* 2003, **49**(3):807-821.
21. Moraes PM, Seyffert N, Silva WM, Castro TL, Silva RF, Lima DD, Hirata R, Silva A, Miyoshi A, Azevedo V: **Characterization of the Opp Peptide Transporter of Corynebacterium pseudotuberculosis and Its Role in Virulence and Pathogenicity**. *BioMed research international* 2014, **2014**.
22. Jones MM, Johnson A, Koszelak-Rosenblum M, Kirkham C, Brauer AL, Malkowski MG, Murphy TF: **Role of the Oligopeptide Permease ABC Transporter of Moraxella catarrhalis in Nutrient Acquisition and Persistence in the Respiratory Tract**. *Infection and immunity* 2014, **82**(11):4758-4766.
23. Roth J, Lawrence J, Bobik T: **Cobalamin (coenzyme B12): synthesis and biological significance**. *Annual Reviews in Microbiology* 1996, **50**(1):137-181.
24. Scott A, Roessner C: **Biosynthesis of cobalamin (vitamin B (12))**. *Biochemical Society Transactions* 2002, **30**(4):613-620.
25. Yin L, Bauer CE: **Controlling the delicate balance of tetrapyrrole biosynthesis**. *Philosophical Transactions of the Royal Society of London B: Biological Sciences* 2013, **368**(1622):20120262.
26. Rodionov DA, Vitreschak AG, Mironov AA, Gelfand MS: **Comparative genomics of the vitamin B12 metabolism and regulation in prokaryotes**. *Journal of Biological Chemistry* 2003, **278**(42):41148-41159.
27. Frankenberg N, Moser J, Jahn D: **Bacterial heme biosynthesis and its biotechnological application**. *Applied microbiology and biotechnology* 2003, **63**(2):115-127.
28. Heldt D, Lawrence A, Lindenmeyer M, Deery E, Heathcote P, Rigby S, Warren M: **Aerobic synthesis of vitamin B12: ring contraction and cobalt chelation**. *Biochemical Society Transactions* 2005, **33**(4):815-819.
29. Moore S, Warren M: **The anaerobic biosynthesis of vitamin B12**. *Biochemical Society Transactions* 2012, **40**(3):581.
30. Smith JL: **The physiological role of ferritin-like compounds in bacteria**. *Critical reviews in microbiology* 2004, **30**(3):173-185.
31. Schalk IJ: **Innovation and Originality in the Strategies Developed by Bacteria To Get Access to Iron**. *Chembiochem* 2013, **14**(3):293-294.
32. Trost E, Ott L, Schneider J, Schröder J, Jaenicke S, Goesmann A, Husemann P, Stoye J, Dorella FA, Rocha FS: **The complete genome sequence of Corynebacterium pseudotuberculosis FRC41 isolated from a 12-year-old girl with necrotizing lymphadenitis reveals insights into gene-regulatory networks contributing to virulence**. *BMC genomics* 2010, **11**(1):728.
33. Köster W: **ABC transporter-mediated uptake of iron, siderophores, heme and vitamin B 12**. *Research in microbiology* 2001, **152**(3):291-301.

34. Kunkle CA, Schmitt MP: **Analysis of a DtxR-regulated iron transport and siderophore biosynthesis gene cluster in Corynebacterium diphtheriae**. *Journal of bacteriology* 2005, **187**(2):422-433.
35. Wandersman C, Delepelaire P: **Bacterial iron sources: from siderophores to hemophores**. *Annu Rev Microbiol* 2004, **58**:611-647.
36. Correnti C, Strong RK: **Mammalian siderophores, siderophore-binding lipocalins, and the labile iron pool**. *Journal of Biological Chemistry* 2012, **287**(17):13524-13531.
37. Contreras H, Chim N, Credali A, Goulding CW: **Heme uptake in bacterial pathogens**. *Current opinion in chemical biology* 2014, **19**:34-41.
38. Allen CE, Schmitt MP: **Novel hemin binding domains in the Corynebacterium diphtheriae HtaA protein interact with hemoglobin and are critical for heme iron utilization by HtaA**. *Journal of bacteriology* 2011, **193**(19):5374-5385.
39. Górska A, Sloderbach A, Marszałł MP: **Siderophore–drug complexes: potential medicinal applications of the 'Trojan horse'strategy**. *Trends in pharmacological sciences* 2014, **35**(9):442-449.
40. Sheldon JR, Heinrichs DE: **Recent developments in understanding the iron acquisition strategies of gram positive pathogens**. *FEMS microbiology reviews* 2015:fuv009.
41. Lutkenhaus and J, Addinall S: **Bacterial cell division and the Z ring**. *Annual review of biochemistry* 1997, **66**(1):93-116.
42. Buss J, Coltharp C, Shtengel G, Yang X, Hess H, Xiao J: **A Multi-layered Protein Network Stabilizes the Escherichia coli FtsZ-ring and Modulates Constriction Dynamics**. 2015.
43. Errington J, Daniel RA, Scheffers D-J: **Cytokinesis in bacteria**. *Microbiology and Molecular Biology Reviews* 2003, **67**(1):52-65.
44. El Zoeiby A, Sanschagrin F, Levesque RC: **Structure and function of the Mur enzymes: development of novel inhibitors**. *Molecular microbiology* 2003, **47**(1):1-12.
45. Carballido-López R, Errington J: **A dynamic bacterial cytoskeleton**. *Trends in cell biology* 2003, **13**(11):577-583.
46. Vollmer W, Blanot D, De Pedro MA: **Peptidoglycan structure and architecture**. *FEMS microbiology reviews* 2008, **32**(2):149-167.
47. den Blaauwen T, Andreu JM, Monasterio O: **Bacterial cell division proteins as antibiotic targets**. *Bioorganic chemistry* 2014, **55**:27-38.
